# Supplementary material for: Accurate DNA methylation predictor for C9orf72 repeat expansion alleles in the pathogenic range
Source: HGG Adv. 2025 Sep 29;7(1):100522. doi: 10.1016/j.xhgg.2025.100522 (PMC12552990; doi:10.1016/j.xhgg.2025.100522)
Supplement: Document S1. Figures S1–S5 and Tables S1 and S2 [file mmc1.pdf]

HGGA, Volume *7*

Supplemental information

Accurate DNA methylation predictor

for *C9orf72* repeat expansion alleles

in the pathogenic range

Naren Ramesh, Alexandria Evans, Kevin Wojta, Zhongan Yang, Marco P. Boks, René S. Kahn, Sterre C.M. de Boer, Sven J. van der Lee, Yolande A.L. Pijnenburg, Lianne M. Reus, and Roel A. Ophoff

Supplementary Materials

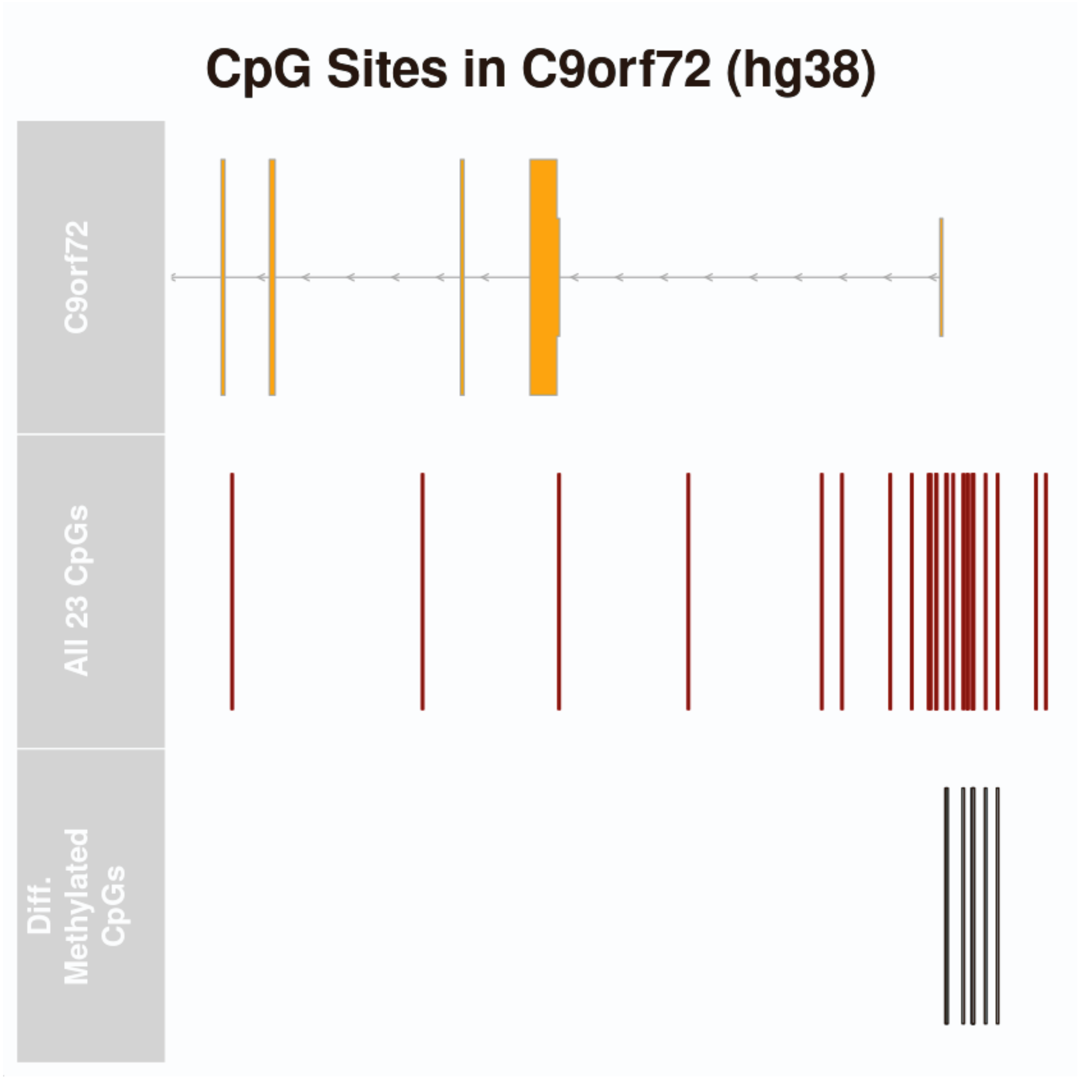

Figure S1: Location of CpGs on *C9orf72* Gene

This plot shows the location relative to the *C9orf72* gene of each of the 23 CpGs included in the model predictive of pathogenic *C9orf72* repeat expansion carrier status as well as the 8 CpGs that were found to be differentially methylated between carriers and non-carriers.

Table S1: Sample Characteristics From Amsterdam Dementia Cohort (ADC)

Characteristic	Carriers of *C9orf72* Expansion > 45 Repeats	Carriers of *C9orf72* Expansion ≤ 45 Repeats	*Adj. P* value
Sample Size (n)	27	250	---
Age, mean (SD)	62.2 (6.7)	63.3 (8.4)	1.00
Female, n (%)	10 (37.0%)	94 (37.6%)	1.00
DNA-methylation Derived Values			
Smoking Score, mean (SD)	-3.54 (3.40)	-3.10 (4.41)	1.00
CD8+ T cells, mean (SD)	0.072 (0.028)	0.083 (0.031)	0.51
CD4+ T cells, mean (SD)	0.174 (0.049)	0.192 (0.049)	0.52
Natural Killer Cells, mean (SD)	0.056 (0.020)	0.058 (0.019)	0.45
B-cells, mean (SD)	0.066 (0.015)	0.071 (0.020)	0.60
Monocytes, mean (SD)	0.055 (0.017)	0.060 (0.023)	1.00
Neutrophils, mean (SD)	0.575 (0.075)	0.536 (0.079)	0.14

This table provides a comparison of key characteristics between carriers and non-carriers of the pathogenic *C9orf72* repeat expansion within the study cohort. The analysis includes DNA methylation-derived measurements such as smoking scores and estimated cell-type proportions, including CD8+ T cells, CD4+ T cells, natural killer cells, B cells, monocytes, and neutrophils.

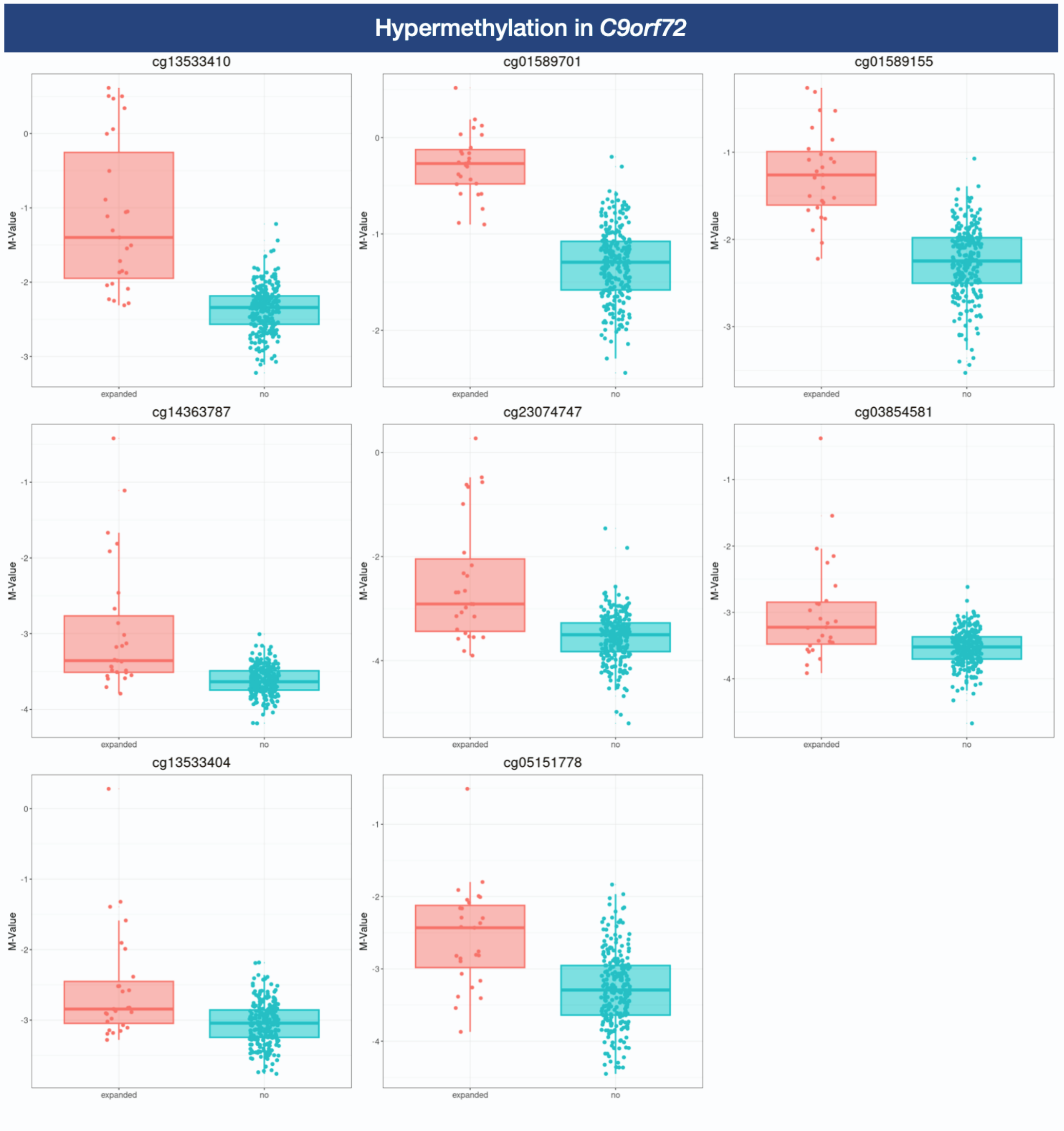

Figure S2: Genome-Wide Significant Differential Methylation Between *C9orf72* Pathogenic Repeat Expansion Carriers and Noncarriers

This figure shows the distribution of M values for the eight CpG sites identified as significantly differentially methylated between carriers and non-carriers of the pathogenic *C9orf72* repeat expansion.

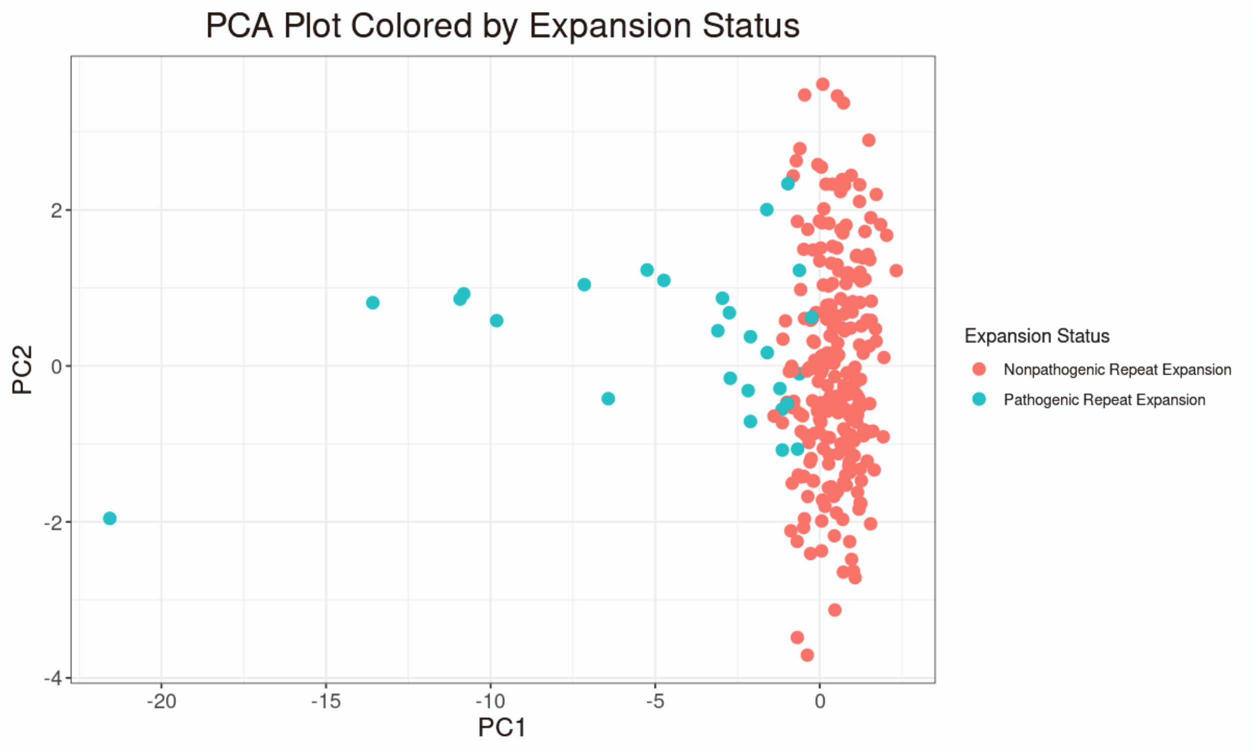

Figure S3: Principal Component 1 vs. 2 of 23 CpG Probes in *C9orf72* Gene Region

This PCA plot compares principal components (PC) 1 and 2 of the CpG probes in the *C9orf72* gene region, colored by whether an individual was a carrier or non-carrier of a pathological *C9orf72* repeat expansion. There is evident separation between individuals with and without pathological *C9orf72* repeat lengths.

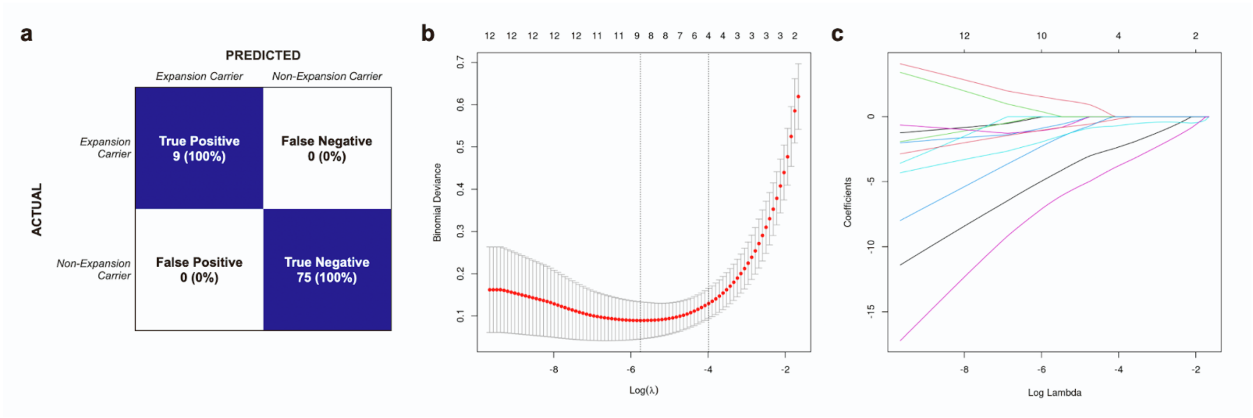

Figure S4: Prediction Confidence Matrix and LASSO Regression Variable Selection

This figure shows the application of Least Absolute Shrinkage and Selection Operator (LASSO) regression to predict pathological *C9orf72* repeat expansion status based on methylation profiles of the 23 CpG probes in the *C9orf72* gene region. (a) Confusion matrix showing 100% accuracy in predicting carrier status in the test set. (b) Cross-validation plot optimizing the lambda parameter to minimize error. (c) Coefficient profiles of the 23 CpG probes as a function of lambda.

Regression Coefficients of CpGs in and 1kb Around *C9Orf72*	
CpG Name	*LASSO Coefficient*
cg13533245	-
cg01126010	-
cg15843044	-
cg01861827	1.4079028
cg13958452	-
cg13533303	-
cg13533310	-
cg13533317	-0.750671
cg13533352	-
cg13533354	-
cg13533361	-0.9740477
cg13533362	-
cg01589155	-4.5619863
cg05151778	-0.9404719
cg05990720	-
cg23074747	-
cg13533397	-
cg11613875	0.1980129
cg03854581	-
cg13533404	-
cg14363787	-1.9280507
cg13533410	-1.7199424
cg01589701	-6.5895417

Table S2: LASSO Regression Coefficients of CpGs in *C9orf72* Gene Region in Initial Model

This table shows the LASSO regression coefficients for CpG probes located within and 1 kb flanking the *C9orf72* gene. Only CpG sites with non-zero coefficients were utilized by the model, and CpGs that were not utilized by the model are labeled as “-”.

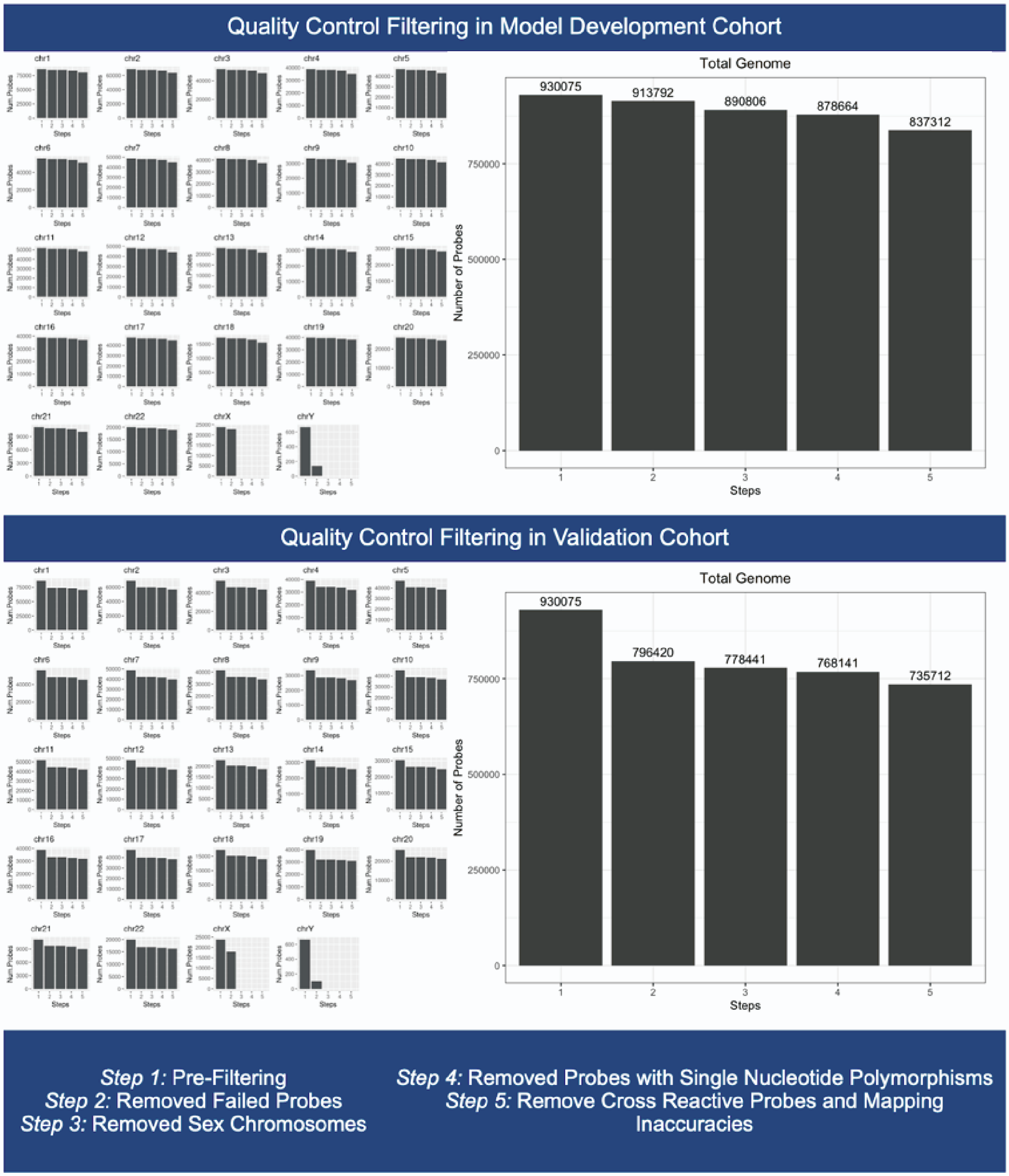

Figure S5: Quality Control Filtering in Both Cohorts

This figure displays the quality control filtering applied to the methylation data in both the model development and validation cohorts. In the model development cohort, probes (930,075) were filtered through five steps: (1) pre-filtering, (2) removal of failed

probes, (3) exclusion of sex chromosome probes, (4) exclusion of probes with single nucleotide polymorphisms (SNPs), and (5) removal of cross-reactive probes and mapping inaccuracies, leaving 837,312 probes. In the validation cohort, similar filtering steps were applied, resulting in 735,712 retained probes after quality control. In both cohorts, each quality control step led to a fairly even distribution of filtering across chromosomes.
