# Supplementary material for: Accurate DNA methylation predictor for C9orf72 repeat expansion alleles in the pathogenic range
Source: HGG Adv. 2025 Sep 29;7(1):100522. doi: 10.1016/j.xhgg.2025.100522 (PMC12552990; doi:10.1016/j.xhgg.2025.100522)
Supplement: Document S2. Article plus supplemental information [file mmc2.pdf]

HGG Advances
ARTICLE

Accurate DNA methylation predictor for *C9orf72* repeat expansion alleles in the pathogenic range

Naren Ramesh,[1] Alexandria Evans,[1] Kevin Wojta,[1] Zhongan Yang,[1] Marco P. Boks,[2,3] René S. Kahn,[4] Sterre C.M. de Boer,[5,6,7] Sven J. van der Lee,[5,6,8] Yolande A.L. Pijnenburg,[5,6] Lianne M. Reus,[5,6] and Roel A. Ophoff[1,9,10,*]

Summary

The hexanucleotide (G_4C_2) repeat expansion in the promoter region of *C9orf72* is the most frequent genetic cause of frontotemporal dementia (FTD) and amyotrophic lateral sclerosis (ALS). In this study, we conducted a genome-wide DNA methylation (DNAm) analysis using EPIC version 2 (EPICv2) arrays on an FTD cohort comprising 27 carriers and 250 non-carriers of the pathogenic *C9orf72* repeat expansion from the Amsterdam Dementia Cohort. We identified differentially methylated CpGs probes associated with the pathogenic *C9orf72* expansion and used these findings to create a DNAm least absolute shrinkage and selection operator (LASSO) predictor to identify repeat expansion carriers. Eight CpG sites at the *C9orf72* locus were significantly differentially hypermethylated in repeat expansion carriers compared to non-carriers. The LASSO model predicted repeat expansion status with an average accuracy of 98.6%. The LASSO predictor was further validated in a separate, independent validation cohort containing 1,589 subjects with bipolar disorder, 580 first-degree relatives, and 289 independent control subjects with available EPICv2 data, identifying four *C9orf72* repeat expansion carriers, subsequently confirmed by repeat-primed PCR. This result highlights the accuracy and generalizability of the DNAm predictor of *C9orf72* repeat expansion carriers. The identification of a highly accurate DNAm biomarker for a repeat expansion locus associated with neurodegenerative disorders may provide great value for studying this locus. The approach holds significant promise for investigating this and other repeat expansion loci, particularly given the growing interest in epigenetic epidemiological studies involving large cohorts with available DNAm data.

Introduction

An increased hexanucleotide repeat expansion of GGGGCC (G_4C_2) in the noncoding region of the *C9orf72* gene is one of the most common genetic causes for amyotrophic lateral sclerosis (ALS) and frontotemporal dementia (FTD).[1–3] This repeat expansion is found in 6%–7% of ALS/FTD without a family history for the disease, 39.3% of familial ALS cases, and 24.8% of familial FTD cases among subjects of European ancestry.[4] While the precise cutoff for non-pathogenic and pathogenic repeat lengths is debated, expansions greater than 30 repeats are generally considered to be pathogenic for ALS and FTD, with some affected individuals having expansions into the thousands of repeat units.[5–7]

Identifying pathogenic *C9orf72* repeat expansions at both the individual and population levels remains crucial. Clinical symptoms associated with *C9orf72* repeat expansion are serious and diverse, with neurodegenerative symptoms manifesting in ALS, FTD, and movement disorders such as parkinsonism but also resulting in behavioral phenotypes observed in FTD and psychiatric-related phenotypes such as schizophrenia and bipolar disorder.[8,9] *C9orf72* expansion is usually tested after suspicion for FTD or ALS or a positive family history for the mutation, but subjects with other disorders and symptoms are not examined for this mutation as part of a standard diagnostic routine.[10] FTD, especially behavioral variant (bvFTD), is often difficult to diagnose or identify due to the heterogeneity of clinical symptoms of the disease, diagnostic overlap with other neurodegenerative disorders, similarity to psychiatric phenotypes, and the lack of biomarkers.[11,12] High percentages of subjects with bvFTD, ranging from 50% to 70%, are misdiagnosed as having other neuropsychiatric conditions or dementia,[13–15] causing an average of 6 years' delay between symptom onset and the diagnosis of bvFTD.[16]

Standard screening for the *C9orf72* repeat expansion is performed using repeat-primed PCR[2] or testing with Southern blotting.[17] Southern blotting allows for more

[1]Center for Neurobehavioral Genetics, Semel Institute for Neuroscience and Human Behavior, University of California, Los Angeles, Los Angeles, CA, USA; [2]Department of Psychiatry, Amsterdam University Medical Center, Amsterdam, the Netherlands; [3]Department of Psychiatry, Brain Center University Medical Center Utrecht, University Utrecht, Utrecht, the Netherlands; [4]Department of Psychiatry, Icahn School of Medicine at Mount Sinai, New York, NY, USA; [5]Alzheimer Center Amsterdam, Neurology, Vrije Universiteit Amsterdam, Amsterdam University Medical Center, Amsterdam, the Netherlands; [6]Amsterdam Neuroscience-Neurodegeneration, Vrije Universiteit Amsterdam, Amsterdam, the Netherlands; [7]The University of Sydney, School of Psychology and Brain & Mind Centre, Sydney, NSW, Australia; [8]Genomics of Neurodegenerative Diseases and Aging, Human Genetics, Vrije Universiteit Amsterdam, VU University Medical Center Amsterdam, Amsterdam, the Netherlands; [9]Department of Human Genetics, David Geffen School of Medicine, University of California, Los Angeles, Los Angeles, CA, USA
[10]Lead contact
*Correspondence: ophoff@g.ucla.edu
https://doi.org/10.1016/j.xhgg.2025.100522.
© 2025 The Author(s). Published by Elsevier Inc. on behalf of American Society of Human Genetics.
This is an open access article under the CC BY license (http://creativecommons.org/licenses/by/4.0/).

precise assessment of the length of the repeat expansion, but the approach is very labor intensive, hard to scale, and requires large amounts of genomic DNA. PCR-based methods, however, are well established for the detection of expanded alleles, can be used in higher-throughput settings, but are not suitable for estimating the actual repeat length itself. More recently, computational methods for the analysis of whole-genome sequence data, such as ExpansionHunter,[18] have become available for the detection of repeat expansions, but the availability of sequence data remains a rate-limiting step. In the near future, we expect that technological advances with long-read sequencing will further advance molecular assessment of *C9orf72* repeat expansion alleles.[19]

Epigenetic features provide valuable insights into the mechanisms underlying *C9orf72* pathogenesis and may help address challenges related to identifying *C9orf72* expansions. Notably, studies have shown that individuals with FTD and ALS often exhibit hypermethylation in the *C9orf72* gene promoter and within the repeat expansion itself.[20,21] Building on these findings, we conducted a genome-wide analysis of differential DNA methylation (DNAm) profiles in subjects with FTD from the Amsterdam Dementia Cohort (ADC) with both pathogenic and non-pathogenic *C9orf72* repeat alleles to assess the extent of DNAm changes associated with these expansions. We identified significant evidence for epigenetic *cis* effects of the repeat expansion and used this insight to develop an accurate DNAm predictor for the detection of pathogenic repeat expansion alleles at this locus.

Subjects and methods

Cohorts

Patients with FTD ($n = 318$) and age- and sex-matched controls ($n = 122$) were included from the ADC for DNAm measurement from whole blood.[22] All subjects and subject controls were of European descent. Six of the 440 samples were flagged as having a sample call rate error, and 157 individuals had no *C9orf72* repeat length data available, leaving 277 in the final test dataset (all subjects with FTD, mean age 63.2 [8.3] years, 37.5% female). The ADC started collecting samples in 2000, and this is an ongoing observational follow-up study of affected individuals who visited the memory clinic of the Alzheimer Center Amsterdam, Amsterdam University Medical Center. All subjects from the ADC underwent a standardized multidisciplinary assessment, consisting of medical history, informant-based history, neurological and medical examination, neuropsychological investigation, electroencephalography, brain magnetic resonance imaging, standard laboratory workup, and lumbar puncture. FTD was diagnosed according to diagnostic guidelines for FTD.[23] A separate, independent cohort was used for validation. This independent validation cohort includes 2,458 individuals of European ancestry (mean age of 51.1 [13.7] years, 57.3% female) without prior evidence of FTD or ALS. This sample consists of $n = 1{,}589$ subjects with bipolar I disorder, $n = 289$ independent controls, and $n = 580$ first-degree relatives of the subjects. This study was performed in accordance with the ethical standards as laid down in the 1964 Declaration of Helsinki and its later amendments. Written informed consent was obtained from all participants.

Repeat-primed PCR screening of the *C9orf72* repeat expansion

The measurement of the GGGGCC repeat of C9orf72 in the discovery cohort has been described in detail elsewhere.[24] PCR validation in the replication cohort was performed as described before.[25] In short, we used both fluorescent and repeat-primed PCR. Fragment length analysis was performed on an ABI 3730/ABI3730xI/3500 genetic analyzer (Applied Biosystems, USA), and data were analyzed using the GeneScan software (version 4/5, ABI) and Peak Scanner software, including a positive control sample for reference.

DNAm measurement and preprocessing

DNA was extracted from whole blood using standardized protocols. A total of 500 ng of each DNA sample was bisulfite converted with an EZ DNAm kit (Zymo Research, USA) following the manufacturer's protocol specifically for a downstream analysis with an Infinium Methylation Microarray. Genome-wide methylation profiles were obtained using an Infinium MethylationEPIC version 2 BeadChip Kit (Illumina, USA) according to the manufacturer's protocol.

A similar preprocessing pipeline was used for both the test dataset and the replication dataset. All data processing was performed using R version 4.0.3. In the test dataset with FTD cases, six samples were removed for failing various technical criteria such as poor bisulfite conversions and poor hybridization or for having low fluorescence intensities. In the validation dataset, six samples were removed for the same criteria.

In both datasets, the following probes were removed: probes with low detection *p* values, probes on sex chromosomes, probes with SNPs[26] at the CG or single-base extension position, cross-reactive probes,[27] and probes flagged by Illumina as problematic in the EPICv2 (EPIC version 2) platform. In both datasets, probe removal was evenly distributed across chromosomes (Figure S5). After a stringent preprocessing procedure, 434 individuals with 837,312 CpG probes remained in the test dataset and 2,458 individuals with 735,712 CpG probes remained in the replication dataset. Since there is no established consensus on using M-values or beta-values for statistical analysis, we opted to use M-values for our analyses.

Differential methylation analysis

A differential methylation analysis was conducted using *limma* to identify differentially methylated probes between subjects with *C9orf72* repeat expansion and subjects without. DNAm values for each CpG probe were regressed against repeat expansion status. Age at sample collection, sex, DNAm-derived cellular composition,[28] DNAm-derived smoking score,[29] and experimental batch were included as covariates for the linear modeling. Genome-wide significance was initially set at $p = 9e{-}8$ in accordance with the literature.[30] However, given the uneven distribution of *C9orf72* pathogenic repeat expansion carriers ($n = 27$) compared to non-carriers ($n = 250$), we conducted a permutation analysis by randomly shuffling carrier status across individuals 100 times to determine the amount of noise that is present in this type of analysis. The most significantly differentially methylated probe in these 100 permutations was subsequently used as

the genome-wide significance threshold (p = 4.4e−10) for this methylation dataset.

Prediction

To predict pathogenic repeat expansions in the *C9orf72* region, we restricted our analysis to the 23 CpG probes located within the *C9orf72* gene as well as 1 kb upstream and downstream of the gene. The data were randomly split into a training set and a test set. The training set comprised 70% of the total set and the test set comprised 30% of the total set. A logistic regression model with L1 regularization was fitted using 10-fold cross-validation using the *glmnet* package on the training data. An initial single model was built to assess accuracy and identify which CpGs were identified as most important. Predictions were made on the test set using the fitted model with the best lambda-value. To assess prediction accuracy across different array technologies, we conducted prediction analyses with the EPICv2 CpGs that were present in EPICv1, Methyl450K, and Methyl27K. For each of the four platforms, we randomly split the data 100 times. Each iteration involved training a least absolute shrinkage and selection operator (LASSO) model on 70% of the data and evaluating its accuracy on the remaining 30% to account for prediction variability due to data splitting.

Independent validation

The initial model trained with CpGs found in EPICv2 was applied to the independent validation dataset. These suspected repeat expansion carriers and 17 random selected subjects were identified as positive and negative controls, respectively.

Results

Sample characteristics

Demographic and clinical characteristics of the ADC cohort are presented in Table S1. For the remainder of this study, we reference expansions that are larger than 45 repeats as pathogenic repeat expansions. This cohort included $n = 27$ FTD subjects with a known pathogenic *C9orf72* repeat expansion (mean age of 62.2 [6.7] years, 37.0% female) and $n = 250$ FTD subjects without a pathogenic *C9orf72* repeat expansion (mean age of 63.3 [8.4] years, 37.6% female). Carrier status of the pathogenic *C9orf72* hexanucleotide repeat expansion was established as part of the diagnostic assessment performed at the Alzheimer Center Amsterdam. Pathological *C9orf72* repeat expansion carriers did not differ from the non-carriers regardless of diagnosis status in terms of age (p = 1.00), sex (p = 1.00), and epigenetic smoking score (p = 1.00).[29] Based on imputed cell composition,[28] pathological *C9orf72* repeat expansion carriers did not differ from the non-carriers in terms of $CD8^+$ T cells (p = 0.51), $CD4^+$ T cells (p = 0.52), natural killer cells (p = 0.45), B cells (p = 0.60), monocytes (p = 1.00), and neutrophils (p = 0.14).

Genome-wide differential methylation analysis of *C9orf72* expansion status

To identify the epigenetic signature of pathological *C9orf72* repeat expansions, we conducted a genome-wide differential methylation analysis on individuals with and without a pathological *C9orf72* repeat expansion, correcting for age, sex, cellular composition, epigenetic smoking score, and experimental batch (n = 277 subjects, 837,312 CpG probes each). We identified eight CpG probes that exhibit differential methylation patterns between individuals with and without pathological *C9orf72* repeat expansions that exceed the genome-wide significance threshold (p = 4.4e−10) (Figure 1A; Table 1). This significance threshold was determined following a permutation analysis randomly shuffling expansion carrier status (of 27 subjects) 100 times. All eight CpGs were hypermethylated in the pathological repeat expansion carriers (Figures 1B, 1C, and S2). We did not find any evidence for genomic inflation of the genome-wide signal (λ_{gc} = 1.006) suggesting that the observed associations are not due to systematic biases or confounding factors (Figure 1D). Notably, these eight most significantly differentially methylated probes were located within the *C9orf72* gene region (Figure 1F). These associations highlight the potential biological (and/or clinical) relevance of methylation changes at this locus and may offer utility in identifying pathological *C9orf72* repeat expansion carriers.

Prediction of expansion using CpGs in the *C9orf72* region

We next examined whether we could predict pathological *C9of72* repeat expansion status using methylation patterns of the CpG probes within the *C9orf72* gene. Given differential methylation patterns in probes within the *C9orf72* gene, we specified our analysis to 23 CpG probes that were found 1 kb upstream, 1 kb downstream, and within *C9orf72* itself. Principal components 1 and 2 on these *C9orf72* probes showed separation between individuals with and without pathological *C9orf72* repeat expansions (Figure S3). We further applied LASSO regression to the 23 CpG probes to predict pathological *C9orf72* repeat expansions. The data were divided into a training set (70%) and a test set (30%), and we employed 10-fold cross-validation to optimize the model parameters and prevent model overfitting (Figures S4B and S4C). In the given test and train split, the LASSO regression model achieved 100% accuracy in predicting pathological *C9orf72* repeat expansion status on the test set (Figure S4A). The model selected nine CpG probes as important predictors (Table S2). Five of these probes were found to be significantly differentially methylated between repeat carriers and non-carriers in our initial differential methylation analysis.

Prediction accuracy is dependent on array technology

Next, we aimed to assess whether predicting pathological *C9orf72* repeat expansion could be successful in previous generation Illumina DNAm array types limiting the LASSO model to CpGs present in EPICv1, Methyl450K, and Methyl 27K. Of the initial 23 CpG probes from

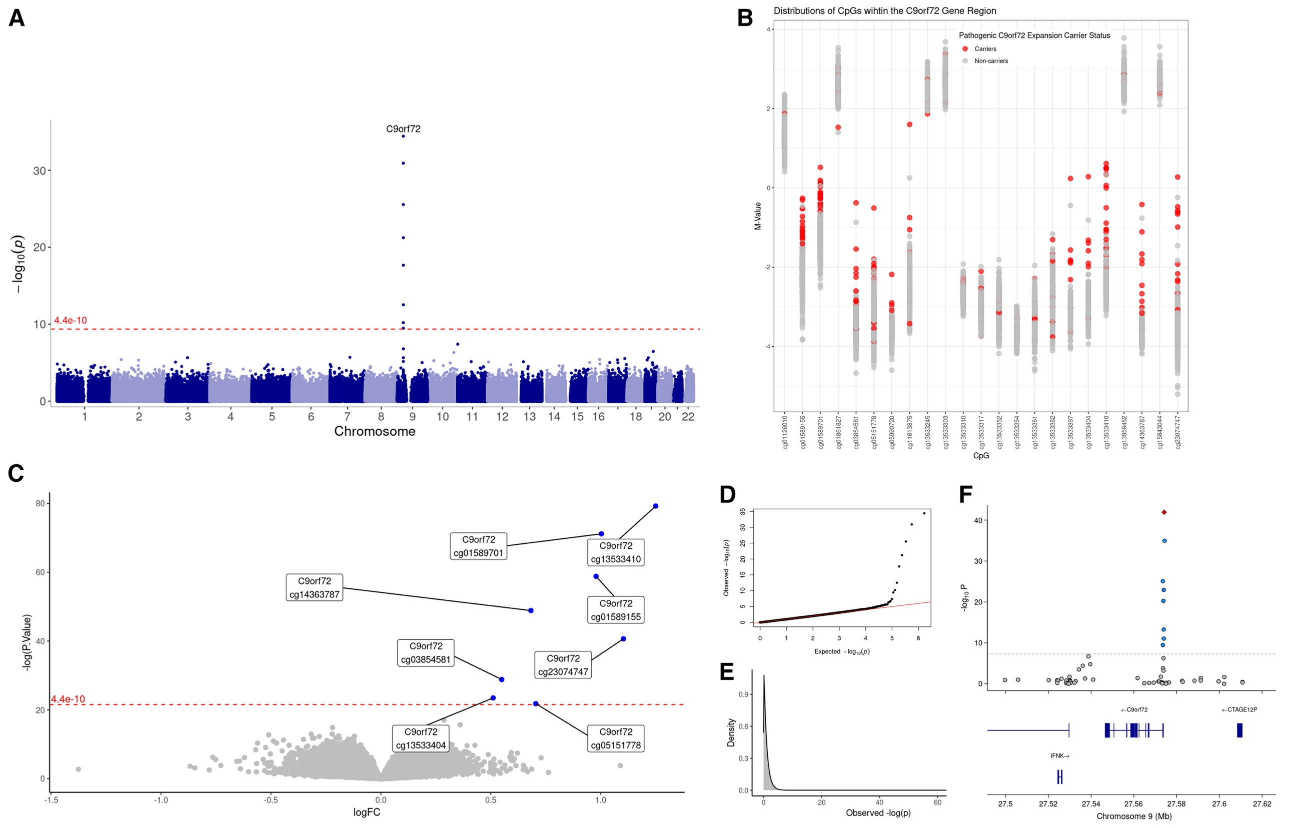

Figure 1. Hypermethylation in the *C9orf72* gene associated with pathological *C9orf72* repeat expansion carriers

(A) Manhattan plot showing *p* values for CpGs tested for differential methylation patterns between carriers and non-carriers of *C9orf72* repeat expansions in the pathogenic range. The genome-wide significance threshold derived from a permutation analysis ($p = 4.4e-10$) is marked by the red dashed line.

(B) Strip plot shows the distribution of M-values of the CpGs in the *C9orf72* gene region (within the gene and 1 kb upstream/downstream). Individuals are colored red if they are carriers of a pathogenic *C9orf72* repeat expansion and colored gray if they are non-carriers.

(C) Volcano plot showing statistical significance of DMA results against log fold change (logFC) illustrating direction of significance. Blue dots represent significant CpGs that are hypermethylated in carriers of *C9orf72* pathogenic repeat expansions. The genome-wide significance threshold derived from a permutation analysis ($p = 4.4e-10$) is marked by the red dashed line.

(D) Quantile-quantile plot showing the *p* value distribution and inflation (λ_{gc} value).

(E) Density plot showing the observed *p* value distribution.

(F) Locus zoom plot of chromosome 9 between 27.5 and 27.62 Mb, including genes in the area. Blue dots indicate significantly differentially methylated CpG probes, and the red diamond indicates the most statistically significant CpG.

EPICv2 within the *C9orf72* gene and 1 kb flanking it, 12 remained in EPICv1, 5 in Methyl450K, and 2 in Methyl27K (Table 2). Using the probes from each platform to predict pathogenic *C9orf72* repeat expansion, we found that the average accuracies were similar between EPICv2 (98.6%) and EPICv1 (99.0%) ($p = 0.059$). However, Methyl450K (94.1%) and Methyl27K (91.7%) were significantly less accurate compared to EPICv2 and EPICv1 (all $p < 1e-15$) (Figure 2A). Average type I error rates were statistically similar in EPICv2 (0.1%), EPICv1 (0.09%), and Methyl450K (0.2%) (all $p > 0.1$). However, type I error rates were significantly higher for Methyl27K (0.7%) compared to Methyl450K ($p = 2.5e-7$), EPICv1 ($p = 2.1e-10$), and EPICv2 ($p = 1.9e-9$) (Figure 2B). Average type II error rates were only nominally different between EPICv2 (11.4%) and EPICv1 ($p = 0.028$). Methyl450K (66.6%) and Methyl27K (75%) had significantly higher average type II rates (Figure 2C). Predicting pathological expansion status using CpG probes from EPICv2 and EPICv1 yielded high accuracy with low false positive and false negative rates. However, using probes from Methyl450K resulted in markedly lower accuracy with a significantly elevated false negative rate. The Methyl27K array had the lowest accuracy, with significantly elevated false positive and false negative rates.

Replication in independent validation cohort

To assess whether these findings could be generalized to other cohorts, we validated our predictor in an independent DNAm cohort for the study of bipolar disorder, a sample that was readily available in our research group. The initial LASSO model from our prior analyses was applied to this independent cohort, and we identified four subjects with a predicted pathological *C9orf72* repeat

Table 1. Differentially methylated CpG probes in *C9orf72* pathogenic repeat expansion carriers

CpG name	Chromosome	Position	Relation to island	Gene	*p* value	Adjusted *p* value	logFC
cg13533410	9	27574186	shore	*C9orf72*	3.78e−35	3.17e−29	57.317367
cg01589701	9	27574385	shore	*C9orf72*	1.22e−31	5.11e−26	51.042004
cg01589155	9	27573534	shore	*C9orf72*	2.90e−26	8.09e−21	41.282198
cg14363787	9	27573988	island	*C9orf72*	5.97e−22	1.25e−16	33.331041
cg23074747	9	27573819	island	*C9orf72*	2.19e−18	3.67e−13	26.687482
cg03854581	9	27573968	island	*C9orf72*	3.03e−13	4.23e−8	17.015665
cg13533404	9	27573982	island	*C9orf72*	6.38e−11	7.63e−6	12.617987
cg05151778	9	27573550	shore	*C9orf72*	3.40e−10	3.56e−5	11.241572

This table presents the CpG probes with significant differential methylation between carriers of the *C9orf72* pathogenic repeat expansion and non-carriers using a significance threshold derived from a permutation analysis ($p = 4.4 \times 10^{-10}$). Each CpG site is annotated with its chromosomal location, relationship to CpG islands, associated gene, and statistical parameters derived from the differential methylation analysis, including the unadjusted *p* value, adjusted *p* value, and log-transformed fold change (FC).

expansion status. Two expansion carriers were subjects with a diagnosis of bipolar disorder (one male and one female in their 50s, both with age of onset of bipolar disorder before age 30), and two other carriers were among the first-degree relatives without a psychiatric or neurological diagnosis (recruited in their mid-50s and early 60s). We selected these 4 individuals and 17 randomly selected individuals of the cohort for independent experimental validation of their *C9orf72* repeat allele status by fluorescent repeat-primed PCR. The validation experiment was performed blindly to the predicted expansion status of these subjects. None of the 17 randomly selected individuals were identified by PCR as having pathological *C9orf72* repeat expansions, while all four predicted pathogenic repeat expansion carriers were indeed confirmed for having an expanded *C9orf72* repeat alleles in the pathogenic range (Figure 3).

Discussion

We performed a genome-wide differential DNAm analysis among carriers and non-carriers of pathogenic *C9orf72* repeat expansions in an FTD cohort. We identified eight CpG probes within the *C9orf72* gene region in pathogenic repeat expansion carriers that are uniquely and significantly hypermethylated after correction for multiple testing. Further analysis showed that DNAm data as obtained from the EPICv2 array can be used to accurately predict individual carrier status of *C9orf72* repeat in the pathogenic range. When applied to an independent DNAm dataset of roughly 2,500 subjects without diagnosis of ALS or FTD, the predictive model identified 4 carriers whose findings were validated by repeat-primed PCR. We observed an accurate prediction for *C9orf72* repeat expansion alleles in the pathogenic range with DNAm analysis, which may serve as a biomarker for epigenetic epidemiological studies with potential clinical relevance for ALS, FTD, and related phenotypes.

Identifying hypermethylation in eight CpG probes in the *C9orf72* gene for pathogenic repeat expansions is consistent with previous studies of ALS and FTD, identifying hypermethylation in the gene promoter region and the repeat itself.[20,21] Hypermethylation has been noted in other GC-rich repeat expansions. For example, the CGG repeat expansion in the X-linked *FMR1* gene associated with fragile X syndrome has been linked with hypermethylation at the promoter region and epigenetic silencing.[31,32] Additionally, the GGC repeat expansion in the *XLT1* gene associated with Baratela-Scott syndrome exhibited hypermethylation in the gene's promoter region.[33] Our genome-wide DNAm analysis results align with the current understanding of hypermethylation around the *C9orf72* repeat expansion being a neuroprotective mechanism to silence pathogenic gene expression.[34,35] It may be that the degree of hypermethylation of this locus in whole blood is clinically relevant for ALS/FTD disease status or progression, but further study is required to examine this in more detail. However, the primary effect of ALS/FTD pathology is neuronal, which may not be detectable in whole blood.

Extending upon our differential methylation findings, the CpG probes at the *C9orf72* locus effectively predict the presence of repeat expansion alleles in the pathogenic range. However, these results were not generalizable across currently available DNAm array platforms. Restricting the model to include CpGs only present on the EPICv1 array did not affect accuracy, but limiting CpGs to those only present in the previous Infinium array types (i.e., Methyl450K and Methyl27K) resulted in significantly diminished accuracy and elevated type I and II error rates. These results indicate that predicting *C9orf72* carrier status only appears to be effective using CpGs present on the higher-resolution EPIC generation of DNAm arrays. Therefore, we recommend this prediction tool be used in DNAm cohorts assayed using EPICv1 and EPICv2.

Our DNAm predictor identified *C9orf72* repeat expansion carriers in a separate independent cohort, a finding

Table 2. **CpG probes in *C9orf72* gene region and a 1-kb flanking region upstream and downstream, across various methylation platforms**

Chromosome	Position	EPICv2	EPICv1	Methyl450K	Methyl27K
9	27561767	cg13533245	–	–	–
9	27564906	cg01126010	cg01126010	–	–
9	27567156	cg15843044	cg15843044	–	–
9	27569287	cg01861827	cg01861827	–	–
9	27571486	cg13958452	cg13958452	cg13958452	–
9	27571819	cg13533303	–	–	–
9	27572615	cg13533310	–	–	–
9	27572969	cg13533317	–	–	–
9	27573249	cg13533352	–	–	–
9	27573281	cg13533354	–	–	–
9	27573369	cg13533361	–	–	–
9	27573379	cg13533362	–	–	–
9	27573534	cg01589155	cg01589155	–	–
9	27573550	cg05151778	cg05151778	–	–
9	27573652	cg05990720	cg05990720	cg05990720	–
9	27573819	cg23074747	cg23074747	cg23074747	cg23074747
9	27573885	cg13533397	–	–	–
9	27573891	cg11613875	cg11613875	cg11613875	cg11613875
9	27573968	cg03854581	cg03854581	–	–
9	27573982	cg13533404	–	–	–
9	27573988	cg14363787	cg14363787	cg14363787	–
9	27574186	cg13533410	–	–	–
9	27574385	cg01589701	cg01589701	–	–

The EPICv2 platform had 23 probes, the EPICv1 platform had 12 probes, the Methyl450K platform had 5 probes, and the Methyl27K platform had 2 probes.

that was thoroughly validated using repeat-primed PCR, a gold standard in *C9orf72* repeat expansion screening. The second cohort is part of an ongoing epigenetic study of severe mental illness with bipolar disorder cases, controls, and first-degree relatives. Of the four individuals identified as having a *C9orf72* repeat expansion in the pathogenic range, two were diagnosed with bipolar disorder and the other two were unaffected siblings of a subject with bipolar disorder. There is no clinical evidence of ALS or FTD diagnosis or family history among these subjects, and in-person clinical re-evaluation of these individuals is not possible. The link between *C9orf72* repeat expansions and psychiatric symptoms, however, remains a topic of interest,[9,36] as are the potential pleiotropic effects of repeat expansions in the pathogenic range in non-FTD or -ALS subjects. The ability to identify carriers in epigenetic epidemiological study cohorts provides new opportunities to examine this further. We observe that the model's complete accuracy in an entirely different cohort of individuals indicates that the model is not overfitted to the initial dataset, strongly validates the robustness and reliability of the model, and paves the way for its integration into other cohorts and broader research.

In the broad context of epigenetics, there is a growing body of research examining the relationship between methylation and its relationship to complex disorders. For instance, one recent study utilized a genome-wide DNAm analysis to identify DNAm variants (also known as epi-variants) at a single locus for neurodevelopmental and congenital anomalies that fail to be explained by conventional genetic testing. This analysis identified epi-variants that were associated with trinucleotide repeat expansion disorders.[37] In another study, researchers found upstream methylation that correlated with the length of the GAA repeat expansion in the first intron of *FXN*, a repeat expansion that results in Friedreich ataxia. These methylation patterns enabled the prediction of expression of *FXN* and clinical outcomes in subjects.[38] Our research adds to this growing body of literature in providing a DNAm-derived tool for predicting repeat expansion status. To our knowledge, our work is one of the first to create a tool to predict individual repeat expansion status.

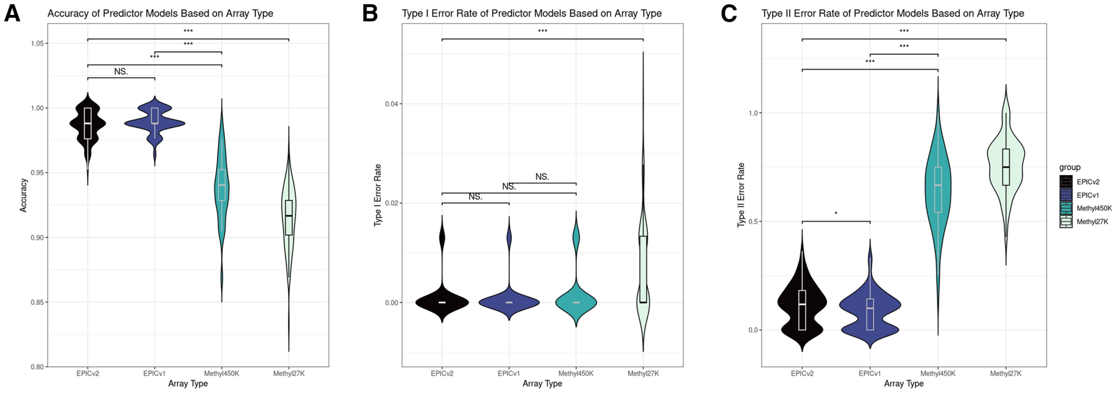

Figure 2. Accuracy, type I error, and type II error across array type
Violin plots showing the distribution of (A) accuracy, (B) type I error (false positive) rates, and (C) type II error (false negative) rates from 100 iterations of training and testing a LASSO model using EPICv2 CpGs present in EPICv2 (23), EPICv1 (12), Methyl450K (5), and Methyl27K (2). NS., not statistically significant.

Epigenetic biomarkers have vastly broadened our understanding of aging. Machine-learning algorithms have been employed to build epigenetic measures of aging using tens to hundreds of CpGs across the genome called epigenetic clocks.[39,40] Clocks have also been trained to reflect age-related covariates like mortality and morbidity.[41,42] For example, GrimAge is an epigenetic clock that combines DNAm-derived values like plasma protein estimates, smoking, age, and sex to determine mortality risk.[43] While these clocks have been effective in predicting a variety of age-related variables, predicting cognitive impairment remains a challenge for many current epigenetic clocks. Some newer clocks like DUNEDinPACE[44] have shown associations with certain dementia-related metrics like Alzheimer disease screening tests and some cognitive tests, but there remains a significant need for reliable measures of biological aging related to dementia and neurological diseases.[45] Integrating DNAm-derived repeat expansions known to cause neurological conditions may offer additional granularity that improves the performance of epigenetic clocks in relation to dementia and other neurological disorders. This pathogenic *C9orf72* repeat expansion predictor may be the first of multiple DNAm-based repeat expansion predictors that aid in improving the performance of these dementia-/cognition-based epigenetic clocks.

Predicting pathogenic repeat expansions in the *C9orf72* gene can offer significant utility in epidemiological studies. This could be of relevance in large-scale schizophrenia and bipolar disorder cohorts, since subjects with *C9orf72*-FTD can often present neuropsychiatric symptoms like mania and psychosis in the early stages of the disease prior to an official diagnosis.[46] Notably, recent population-level analyses have shown that the penetrance of pathogenic *C9orf72* repeat expansions may be as low as 33% in ALS, suggesting that a substantial proportion of carriers may remain clinically unaffected.[47] However, given the phenotypic heterogeneity of FTD, awareness of individuals with *C9orf72* pathological repeat expansion *a priori* in any large methylation dataset with subjects with neurobehavioral conditions is extremely valuable.[48] Accurately identifying subjects with bvFTD in the early stages is crucial for future FTD clinical trials.[10] We identified four C9orf72 repeat expansion carriers among the 1,589 subjects with bipolar disorder and 580 first-degree relatives, indicating a prevalence of 0.18%. This is consistent with what is reported in other studies (i.e., 0.151% in the European global population[49] and 0.232% in subjects with bipolar disorder[46]).

There are some limitations to this study. First, our dataset is small, and there are far more individuals without than subjects with the pathogenic repeat expansion. However, the strength of the differential methylation analysis significant hits, given the size of this cohort, shows how strong and clear the association between the eight *C9orf72* CpGs and pathogenic repeat expansion status is. Second, these findings are limited to whole-blood DNA samples, which may not reflect the brain epigenetic patterns implicated in neurological conditions. Differing observations regarding somatic heterogeneity, referring to variation of the *C9orf72* repeat expansion size within an individual between tissue types, have been made in prior studies. One study observed somatic heterogeneity in individuals with large *C9orf72* repeat expansions, while another study found that *C9orf72* repeat expansion is consistent between brain and blood in asymptomatic or affected pathogenic *C9orf72* repeat expansion carriers.[17,20] Third, both this cohort and the independent cohort used for validation contain subjects of European ancestry. The large *C9orf72* repeat expansion haplotype does seem to have a common European founder, but additional research needs to be done to see whether these results hold in subjects of varying ethnic and ancestral backgrounds.[8,50]

In summary, the epigenetic *C9orf72* repeat expansion predictor offers significant utility at an individual clinical level, at a population level for large DNAm cohorts, and at a research level, for study of neurodegenerative disorders, of pleiotropic effects of *C9orf72* repeat expansions,

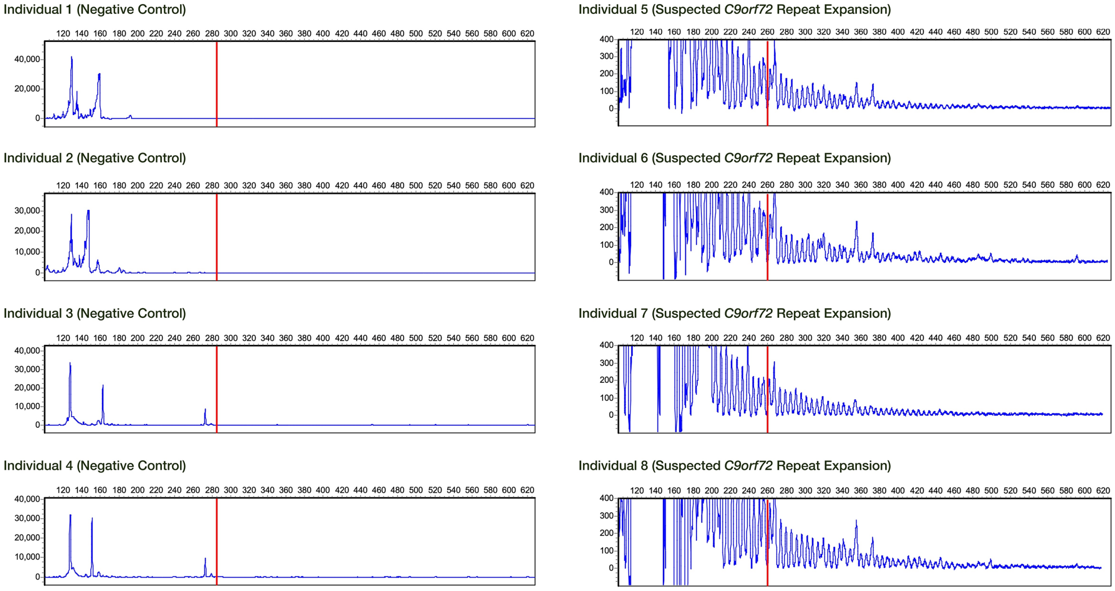

Figure 3. PCR plots from four randomly selected negative controls and four suspected pathogenic *C9orf72* repeat expansion carriers in the replication cohort
PCR plots from 4 of the 17 randomly selected negative controls from the validation cohort (individuals 1–4) and 4 pathogenic *C9orf72* repeat expansion carriers identified by LASSO model (individuals 5–8). The red line denotes the cutoff for what is considered to be a pathogenic repeat expansion length.

epigenetic aging, and neurological and cognitive outcomes. We expect that other repeat expansions and types of genomic variation are also detectable via epigenetic profiling and that this DNAm predictor will be a first among others to be used clinically and in research.

Data and code availability

All analysis scripts generated during this study are available at GitHub (github.com/naren-ramesh/C9Orf72-Expansion-Prediction). There are restrictions to the availability of original methylation array data and clinical files due to subject privacy concerns. Cleaned M-values for CpGs located within ±1 kb of the *C9orf72* gene for both the training and independent validation cohorts are provided on GitHub.

Acknowledgments

The research of the Alzheimer Center Amsterdam is part of the neurodegeneration research program of Amsterdam Neuroscience. Alzheimer Center Amsterdam is supported by Stichting Alzheimer Nederland and Stichting Steun Alzheimercentrum Amsterdam. The clinical database structure was developed with funding from Stichting Dioraphte. We are greatly appreciative to the individuals who donated the blood samples on which this study was based. L.M.R. was funded by the Memorabel fellowship (ZonMw project no. 10510022110012). The FTD DNAm study was supported by the National Institutes of Health/National Institute on Aging (NIH/NIA) grant no. R21 AG072390.

Declaration of interests

The authors declare no competing interests.

Supplemental information

Supplemental information can be found online at https://doi.org/10.1016/j.xhgg.2025.100522.

Received: April 9, 2025
Accepted: September 25, 2025

References

1. Renton, A.E., Majounie, E., Waite, A., Simón-Sánchez, J., Rollinson, S., Gibbs, J.R., Schymick, J.C., Laaksovirta, H., van Swieten, J.C., Myllykangas, L., et al. (2011). A hexanucleotide repeat expansion in C9ORF72 is the cause of chromosome 9p21-linked ALS-FTD. Neuron *72*, 257–268.
2. DeJesus-Hernandez, M., Mackenzie, I.R., Boeve, B.F., Boxer, A.L., Baker, M., Rutherford, N.J., Nicholson, A.M., Finch, N.A., Flynn, H., Adamson, J., et al. (2011). Expanded GGGGCC hexanucleotide repeat in noncoding region of C9ORF72 causes chromosome 9p-linked FTD and ALS. Neuron *72*, 245–256.
3. Gijselinck, I., Van Langenhove, T., van der Zee, J., Sleegers, K., Philtjens, S., Kleinberger, G., Janssens, J., Bettens, K., Van Cauwenberghe, C., Pereson, S., et al. (2012). A C9orf72 promoter repeat expansion in a Flanders-Belgian cohort with disorders of the frontotemporal lobar degeneration-amyotrophic lateral sclerosis spectrum: a gene identification study. Lancet Neurol. *11*, 54–65.
4. Majounie, E., Renton, A.E., Mok, K., Dopper, E.G.P., Waite, A., Rollinson, S., Chiò, A., Restagno, G., Nicolaou, N., Simon-Sanchez, J., et al. (2012). Frequency of the C9orf72 hexanucleotide repeat expansion in patients with amyotrophic lateral sclerosis and frontotemporal dementia: a cross-sectional study. Lancet Neurol. *11*, 323–330.

5. Van Mossevelde, S., van der Zee, J., Cruts, M., and Van Broeckhoven, C. (2017). Relationship between C9orf72 repeat size and clinical phenotype. Curr. Opin. Genet. Dev. *44*, 117–124.
6. Gijselinck, I., Van Mossevelde, S., van der Zee, J., Sieben, A., Engelborghs, S., De Bleecker, J., Ivanoiu, A., Deryck, O., Edbauer, D., Zhang, M., et al. (2016). The C9orf72 repeat size correlates with onset age of disease, DNA methylation and transcriptional downregulation of the promoter. Mol. Psychiatry *21*, 1112–1124.
7. Gijselinck, I., Cruts, M., and Van Broeckhoven, C. (2018). The Genetics of C9orf72 Expansions. Cold Spring Harb. Perspect. Med. *8*, a026757. https://doi.org/10.1101/cshperspect.a026757.
8. Breevoort, S., Gibson, S., Figueroa, K., Bromberg, M., and Pulst, S. (2022). Expanding Clinical Spectrum of C9ORF72-Related Disorders and Promising Therapeutic Strategies: A Review. Neurol. Genet. *8*, e670.
9. Silverman, H.E., Goldman, J.S., and Huey, E.D. (2019). Links Between the C9orf72 Repeat Expansion and Psychiatric Symptoms. Curr. Neurol. Neurosci. Rep. *19*, 93.
10. Ducharme, S., Dols, A., Laforce, R., Devenney, E., Kumfor, F., van den Stock, J., Dallaire-Théroux, C., Seelaar, H., Gossink, F., Vijverberg, E., et al. (2020). Recommendations to distinguish behavioural variant frontotemporal dementia from psychiatric disorders. Brain *143*, 1632–1650.
11. Rohrer, J.D., Isaacs, A.M., Mizielinska, S., Mead, S., Lashley, T., Wray, S., Sidle, K., Fratta, P., Orrell, R.W., Hardy, J., et al. (2015). C9orf72 expansions in frontotemporal dementia and amyotrophic lateral sclerosis. Lancet Neurol. *14*, 291–301.
12. Zampatti, S., Peconi, C., Campopiano, R., Gambardella, S., Caltagirone, C., and Giardina, E. (2022). C9orf72-related neurodegenerative diseases: From clinical diagnosis to therapeutic strategies. Front. Aging Neurosci. *14*, 907122.
13. Tsoukra, P., Velakoulis, D., Wibawa, P., Malpas, C.B., Walterfang, M., Evans, A., Farrand, S., Kelso, W., Eratne, D., and Loi, S.M. (2022). The diagnostic challenge of young-onset dementia syndromes and primary psychiatric diseases: Results from a retrospective 20-year cross-sectional study. J. Neuropsychiatry Clin. Neurosci. *34*, 44–52.
14. Woolley, J.D., Khan, B.K., Murthy, N.K., Miller, B.L., and Rankin, K.P. (2011). The diagnostic challenge of psychiatric symptoms in neurodegenerative disease: rates of and risk factors for prior psychiatric diagnosis in patients with early neurodegenerative disease. J. Clin. Psychiatry *72*, 126–133.
15. de Boer, S.C.M., Gossink, F., Krudop, W., Vijverberg, E., Schouws, S., Reus, L.M., Pijnenburg, Y.A.L., and Dols, A. (2023). Diagnostic instability over time in the late-onset frontal lobe syndrome: When can we say it's FTD? Am. J. Geriatr. Psychiatry *31*, 679–690.
16. van Vliet, D., de Vugt, M.E., Bakker, C., Pijnenburg, Y.A.L., Vernooij-Dassen, M.J.F.J., Koopmans, R.T.C.M., and Verhey, F.R.J. (2013). Time to diagnosis in young-onset dementia as compared with late-onset dementia. Psychol. Med. *43*, 423–432.
17. van Blitterswijk, M., DeJesus-Hernandez, M., Niemantsverdriet, E., Murray, M.E., Heckman, M.G., Diehl, N.N., Brown, P.H., Baker, M.C., Finch, N.A., Bauer, P.O., et al. (2013). Association between repeat sizes and clinical and pathological characteristics in carriers of C9ORF72 repeat expansions (Xpansize-72): a cross-sectional cohort study. Lancet Neurol. *12*, 978–988.
18. Dolzhenko, E., Deshpande, V., Schlesinger, F., Krusche, P., Petrovski, R., Chen, S., Emig-Agius, D., Gross, A., Narzisi, G., Bowman, B., et al. (2019). ExpansionHunter: a sequence-graph-based tool to analyze variation in short tandem repeat regions. Bioinformatics *35*, 4754–4756.
19. Ebbert, M.T.W., Farrugia, S.L., Sens, J.P., Jansen-West, K., Gendron, T.F., Prudencio, M., McLaughlin, I.J., Bowman, B., Seetin, M., DeJesus-Hernandez, M., et al. (2018). Long-read sequencing across the C9orf72 "GGGGCC" repeat expansion: implications for clinical use and genetic discovery efforts in human disease. Mol. Neurodegener. *13*, 46.
20. Xi, Z., Zhang, M., Bruni, A.C., Maletta, R.G., Colao, R., Fratta, P., Polke, J.M., Sweeney, M.G., Mudanohwo, E., Nacmias, B., et al. (2015). The C9orf72 repeat expansion itself is methylated in ALS and FTLD patients. Acta Neuropathol. *129*, 715–727.
21. Xi, Z., Zinman, L., Moreno, D., Schymick, J., Liang, Y., Sato, C., Zheng, Y., Ghani, M., Dib, S., Keith, J., et al. (2013). Hypermethylation of the CpG island near the G4C2 repeat in ALS with a C9orf72 expansion. Am. J. Hum. Genet. *92*, 981–989.
22. van der Flier, W.M., and Scheltens, P. (2018). Amsterdam Dementia Cohort: Performing research to optimize care. J. Alzheimers Dis. *62*, 1091–1111.
23. Rascovsky, K., Hodges, J.R., Knopman, D., Mendez, M.F., Kramer, J.H., Neuhaus, J., van Swieten, J.C., Seelaar, H., Dopper, E.G.P., Onyike, C.U., et al. (2011). Sensitivity of revised diagnostic criteria for the behavioural variant of frontotemporal dementia. Brain *134*, 2456–2477.
24. Reus, L.M., Jansen, I.E., Mol, M.O., van Ruissen, F., van Rooij, J., van Schoor, N.M., Tesi, N., Reinders, M.J.T., Huisman, M.A., Holstege, H., et al. (2021). Genome-wide association study of frontotemporal dementia identifies a C9ORF72 haplotype with a median of 12-G4C2 repeats that predisposes to pathological repeat expansions. Transl. Psychiatry *11*, 451.
25. Ramos, E.M., Dokuru, D.R., Van Berlo, V., Wojta, K., Wang, Q., Huang, A.Y., Deverasetty, S., Qin, Y., van Blitterswijk, M., Jackson, J., et al. (2020). Genetic screening of a large series of North American sporadic and familial frontotemporal dementia cases. Alzheimers Dement. *16*, 118–130.
26. Zhou, W., Laird, P.W., and Shen, H. (2017). Comprehensive characterization, annotation and innovative use of Infinium DNA methylation BeadChip probes. Nucleic Acids Res. *45*, e22.
27. Chen, Y.-A., Lemire, M., Choufani, S., Butcher, D.T., Grafodatskaya, D., Zanke, B.W., Gallinger, S., Hudson, T.J., and Weksberg, R. (2013). Discovery of cross-reactive probes and polymorphic CpGs in the Illumina Infinium HumanMethylation450 microarray. Epigenetics *8*, 203–209.
28. Houseman, E.A., Accomando, W.P., Koestler, D.C., Christensen, B.C., Marsit, C.J., Nelson, H.H., Wiencke, J.K., and Kelsey, K.T. (2012). DNA methylation arrays as surrogate measures of cell mixture distribution. BMC Bioinf. *13*, 86.
29. Bollepalli, S., Korhonen, T., Kaprio, J., Anders, S., and Ollikainen, M. (2019). EpiSmokEr: a robust classifier to determine smoking status from DNA methylation data. Epigenomics *11*, 1469–1486.
30. Mansell, G., Gorrie-Stone, T.J., Bao, Y., Kumari, M., Schalkwyk, L.S., Mill, J., and Hannon, E. (2019). Guidance for DNA methylation studies: statistical insights from the Illumina EPIC array. BMC Genom. *20*, 366.

31. Tabolacci, E., Pomponi, M.G., Remondini, L., Pietrobono, R., Nobile, V., Pennacchio, G., Gurrieri, F., Neri, G., Genuardi, M., and Chiurazzi, P. (2020). Methylated premutation of the FMR1 gene in three sisters: correlating CGG expansion and epigenetic inactivation. Eur. J. Hum. Genet. *28*, 567–575.
32. Nobile, V., Pucci, C., Chiurazzi, P., Neri, G., and Tabolacci, E. (2021). DNA methylation, mechanisms of FMR1 inactivation and therapeutic perspectives for Fragile X syndrome. Biomolecules *11*, 296.
33. LaCroix, A.J., Stabley, D., Sahraoui, R., Adam, M.P., Mehaffey, M., Kernan, K., Myers, C.T., Fagerstrom, C., Anadiotis, G., Akkari, Y.M., et al. (2019). GGC repeat expansion and Exon 1 methylation of XYLT1 is a common pathogenic variant in baratela-Scott syndrome. Am. J. Hum. Genet. *104*, 35–44.
34. Liu, E.Y., Russ, J., Wu, K., Neal, D., Suh, E., McNally, A.G., Irwin, D.J., Van Deerlin, V.M., and Lee, E.B. (2014). C9orf72 hypermethylation protects against repeat expansion-associated pathology in ALS/FTD. Acta Neuropathol. *128*, 525–541.
35. McMillan, C.T., Russ, J., Wood, E.M., Irwin, D.J., Grossman, M., McCluskey, L., Elman, L., Van Deerlin, V., and Lee, E.B. (2015). C9orf72 promoter hypermethylation is neuroprotective: Neuroimaging and neuropathologic evidence. Neurology *84*, 1622–1630.
36. Meisler, M.H., Grant, A.E., Jones, J.M., Lenk, G.M., He, F., Todd, P.K., Kamali, M., Albin, R.L., Lieberman, A.P., Langenecker, S.A., and McInnis, M.G. (2013). C9ORF72 expansion in a family with bipolar disorder. Bipolar Disord. *15*, 326–332.
37. Aref-Eshghi, E., Bend, E.G., Colaiacovo, S., Caudle, M., Chakrabarti, R., Napier, M., Brick, L., Brady, L., Carere, D.A., Levy, M.A., et al. (2019). Diagnostic utility of genome-wide DNA methylation testing in genetically unsolved individuals with suspected hereditary conditions. Am. J. Hum. Genet. *104*, 685–700.
38. Evans-Galea, M.V., Carrodus, N., Rowley, S.M., Corben, L.A., Tai, G., Saffery, R., Galati, J.C., Wong, N.C., Craig, J.M., Lynch, D.R., et al. (2012). FXN methylation predicts expression and clinical outcome in Friedreich ataxia. Ann. Neurol. *71*, 487–497.
39. Horvath, S., and Raj, K. (2018). DNA methylation-based biomarkers and the epigenetic clock theory of ageing. Nat. Rev. Genet. *19*, 371–384.
40. Levine, M.E. (2020). Assessment of epigenetic clocks as biomarkers of aging in basic and population research. J. Gerontol. A Biol. Sci. Med. Sci. *75*, 463–465.
41. Zhang, Y., Wilson, R., Heiss, J., Breitling, L.P., Saum, K.-U., Schöttker, B., Holleczek, B., Waldenberger, M., Peters, A., and Brenner, H. (2017). DNA methylation signatures in peripheral blood strongly predict all-cause mortality. Nat. Commun. *8*, 14617.
42. Levine, M.E., Lu, A.T., Quach, A., Chen, B.H., Assimes, T.L., Bandinelli, S., Hou, L., Baccarelli, A.A., Stewart, J.D., Li, Y., et al. (2018). An epigenetic biomarker of aging for lifespan and healthspan. Aging (Albany NY) *10*, 573–591.
43. Lu, A.T., Quach, A., Wilson, J.G., Reiner, A.P., Aviv, A., Raj, K., Hou, L., Baccarelli, A.A., Li, Y., Stewart, J.D., et al. (2019). DNA methylation GrimAge strongly predicts lifespan and healthspan. Aging (Albany NY) *11*, 303–327.
44. Belsky, D.W., Caspi, A., Corcoran, D.L., Sugden, K., Poulton, R., Arseneault, L., Baccarelli, A., Chamarti, K., Gao, X., Hannon, E., et al. (2022). DunedinPACE, a DNA methylation biomarker of the pace of aging. eLife *11*, e73420. https://doi.org/10.7554/eLife.73420.
45. Sugden, K., Caspi, A., Elliott, M.L., Bourassa, K.J., Chamarti, K., Corcoran, D.L., Hariri, A.R., Houts, R.M., Kothari, M., Kritchevsky, S., et al. (2022). Association of pace of aging measured by blood-based DNA methylation with age-related cognitive impairment and dementia. Neurology *99*, e1402–e1413.
46. Ducharme, S., Bajestan, S., Dickerson, B.C., and Voon, V. (2017). Psychiatric presentations of C9orf72 mutation: What are the diagnostic implications for clinicians? J. Neuropsychiatry Clin. Neurosci. *29*, 195–205.
47. Douglas, A.G.L., and Baralle, D. (2024). Reduced penetrance of gene variants causing amyotrophic lateral sclerosis. J. Med. Genet. *61*, 294–297. https://doi.org/10.1136/jmg-2023-109580.
48. Landqvist Waldö, M., Gustafson, L., Passant, U., and Englund, E. (2015). Psychotic symptoms in frontotemporal dementia: a diagnostic dilemma? Int. Psychogeriatr. *27*, 531–539.
49. Ibañez, K., Jadhav, B., Zanovello, M., Gagliardi, D., Clarkson, C., Facchini, S., Garg, P., Martin-Trujillo, A., Gies, S.J., Galassi Deforie, V., et al. (2024). Increased frequency of repeat expansion mutations across different populations. Nat. Med. *30*, 3357–3368. https://doi.org/10.1038/s41591-024-03190-5.
50. Smith, B.N., Newhouse, S., Shatunov, A., Vance, C., Topp, S., Johnson, L., Miller, J., Lee, Y., Troakes, C., Scott, K.M., et al. (2013). The C9ORF72 expansion mutation is a common cause of ALS+/-FTD in Europe and has a single founder. Eur. J. Hum. Genet. *21*, 102–108.

HGGA, Volume 7

Supplemental information

Accurate DNA methylation predictor for *C9orf72* repeat expansion alleles in the pathogenic range

Naren Ramesh, Alexandria Evans, Kevin Wojta, Zhongan Yang, Marco P. Boks, René S. Kahn, Sterre C.M. de Boer, Sven J. van der Lee, Yolande A.L. Pijnenburg, Lianne M. Reus, and Roel A. Ophoff

Supplementary Materials

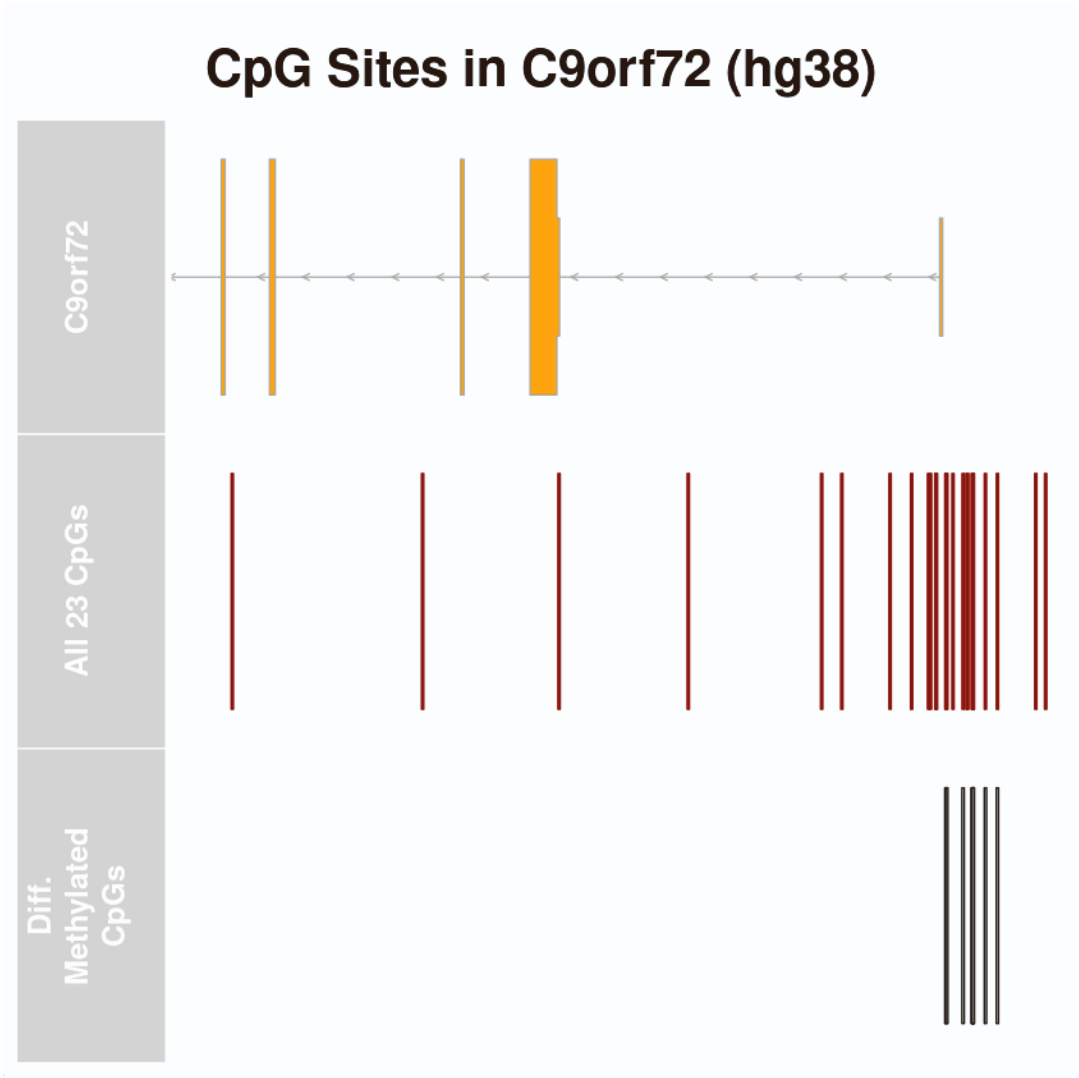

Figure S1: Location of CpGs on *C9orf72* Gene

This plot shows the location relative to the *C9orf72* gene of each of the 23 CpGs included in the model predictive of pathogenic *C9orf72* repeat expansion carrier status as well as the 8 CpGs that were found to be differentially methylated between carriers and non-carriers.

Table S1: Sample Characteristics From Amsterdam Dementia Cohort (ADC)

Characteristic	Carriers of *C9orf72* Expansion > 45 Repeats	Carriers of *C9orf72* Expansion ≤ 45 Repeats	*Adj. P* value
Sample Size (n)	27	250	---
Age, mean (SD)	62.2 (6.7)	63.3 (8.4)	1.00
Female, n (%)	10 (37.0%)	94 (37.6%)	1.00
DNA-methylation Derived Values			
Smoking Score, mean (SD)	-3.54 (3.40)	-3.10 (4.41)	1.00
CD8+ T cells, mean (SD)	0.072 (0.028)	0.083 (0.031)	0.51
CD4+ T cells, mean (SD)	0.174 (0.049)	0.192 (0.049)	0.52
Natural Killer Cells, mean (SD)	0.056 (0.020)	0.058 (0.019)	0.45
B-cells, mean (SD)	0.066 (0.015)	0.071 (0.020)	0.60
Monocytes, mean (SD)	0.055 (0.017)	0.060 (0.023)	1.00
Neutrophils, mean (SD)	0.575 (0.075)	0.536 (0.079)	0.14

This table provides a comparison of key characteristics between carriers and non-carriers of the pathogenic *C9orf72* repeat expansion within the study cohort. The analysis includes DNA methylation-derived measurements such as smoking scores and estimated cell-type proportions, including CD8+ T cells, CD4+ T cells, natural killer cells, B cells, monocytes, and neutrophils.

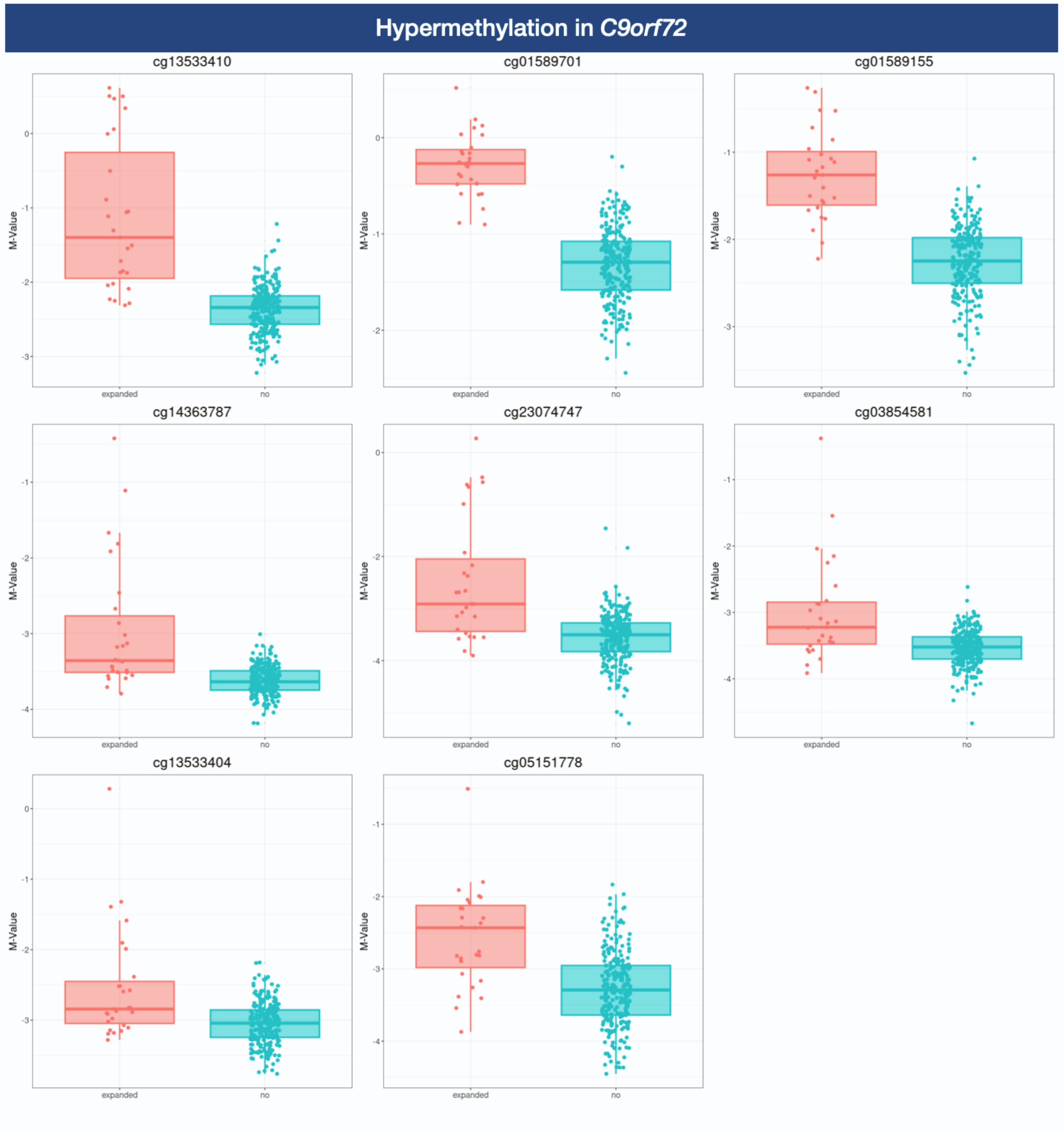

Figure S2: Genome-Wide Significant Differential Methylation Between *C9orf72* Pathogenic Repeat Expansion Carriers and Noncarriers

This figure shows the distribution of M values for the eight CpG sites identified as significantly differentially methylated between carriers and non-carriers of the pathogenic *C9orf72* repeat expansion.

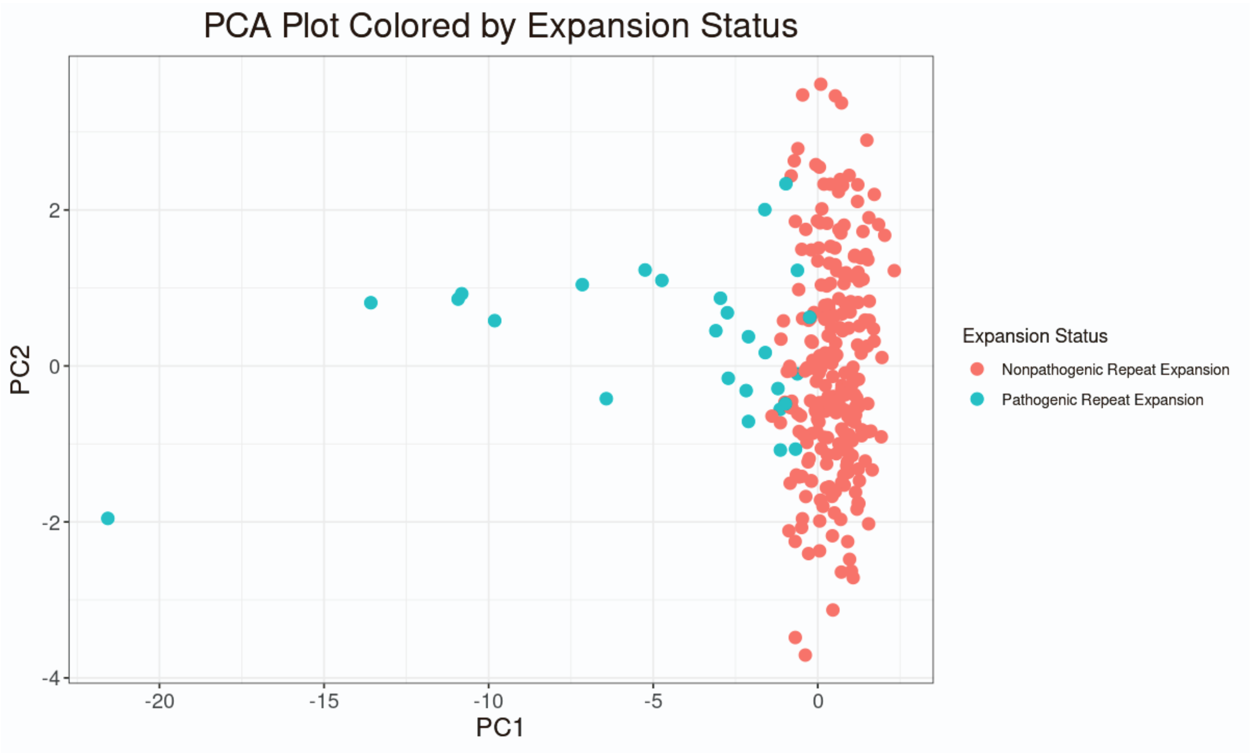

Figure S3: Principal Component 1 vs. 2 of 23 CpG Probes in *C9orf72* Gene Region

This PCA plot compares principal components (PC) 1 and 2 of the CpG probes in the *C9orf72* gene region, colored by whether an individual was a carrier or non-carrier of a pathological *C9orf72* repeat expansion. There is evident separation between individuals with and without pathological *C9orf72* repeat lengths.

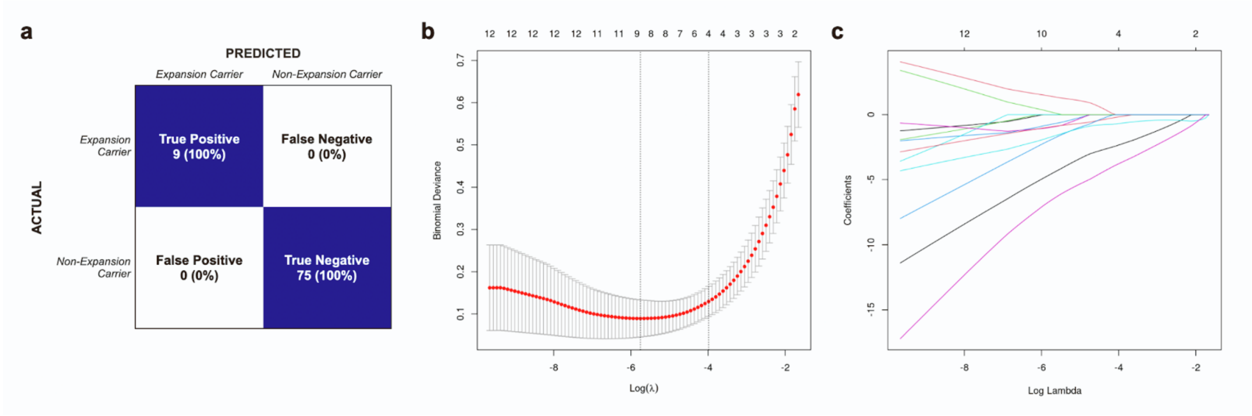

Figure S4: Prediction Confidence Matrix and LASSO Regression Variable Selection

This figure shows the application of Least Absolute Shrinkage and Selection Operator (LASSO) regression to predict pathological *C9orf72* repeat expansion status based on methylation profiles of the 23 CpG probes in the *C9orf72* gene region. (a) Confusion matrix showing 100% accuracy in predicting carrier status in the test set. (b) Cross-validation plot optimizing the lambda parameter to minimize error. (c) Coefficient profiles of the 23 CpG probes as a function of lambda.

Regression Coefficients of CpGs in and 1kb Around *C9Orf72*	
CpG Name	*LASSO Coefficient*
cg13533245	-
cg01126010	-
cg15843044	-
cg01861827	1.4079028
cg13958452	-
cg13533303	-
cg13533310	-
cg13533317	-0.750671
cg13533352	-
cg13533354	-
cg13533361	-0.9740477
cg13533362	-
cg01589155	-4.5619863
cg05151778	-0.9404719
cg05990720	-
cg23074747	-
cg13533397	-
cg11613875	0.1980129
cg03854581	-
cg13533404	-
cg14363787	-1.9280507
cg13533410	-1.7199424
cg01589701	-6.5895417

Table S2: LASSO Regression Coefficients of CpGs in *C9orf72* Gene Region in Initial Model

This table shows the LASSO regression coefficients for CpG probes located within and 1 kb flanking the *C9orf72* gene. Only CpG sites with non-zero coefficients were utilized by the model, and CpGs that were not utilized by the model are labeled as “-”.

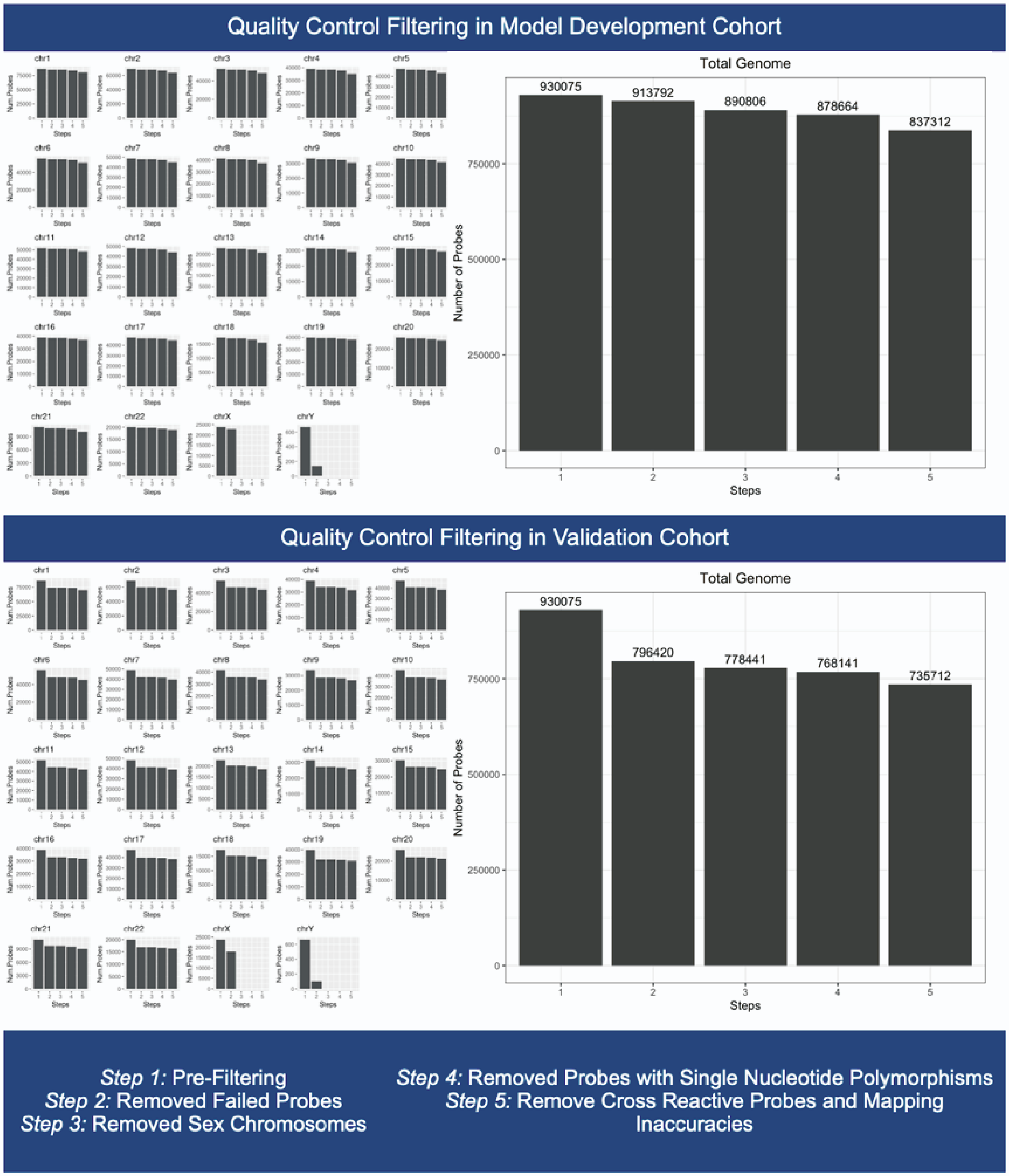

Figure S5: Quality Control Filtering in Both Cohorts

This figure displays the quality control filtering applied to the methylation data in both the model development and validation cohorts. In the model development cohort, probes (930,075) were filtered through five steps: (1) pre-filtering, (2) removal of failed

probes, (3) exclusion of sex chromosome probes, (4) exclusion of probes with single nucleotide polymorphisms (SNPs), and (5) removal of cross-reactive probes and mapping inaccuracies, leaving 837,312 probes. In the validation cohort, similar filtering steps were applied, resulting in 735,712 retained probes after quality control. In both cohorts, each quality control step led to a fairly even distribution of filtering across chromosomes.
